# Downregulation of PIK3IP1 in retinal microglia promotes retinal pathological neovascularization via PI3K-AKT pathway activation

**DOI:** 10.1038/s41598-023-39473-z

**Published:** 2023-08-07

**Authors:** Lushu Chen, Yuan Cao, Yaming Shen, Huan Li, Rong Ye, Jin Yao

**Affiliations:** 1 The Fourth School of Clinical Medicine, Nanjing Medical University, Nanjing, China; 2 The Affiliated Eye Hospital, Nanjing Medical University, 138 Han Zhong Road, Nanjing, 210029 China

**Keywords:** Retinal diseases, Immunology, Inflammation

## Abstract

Retinal pathological neovascularization involves endothelial cells, pericytes, photoreceptor cells, ganglion cells, and glial cells, whose roles remain unclear. Using the Scissor algorithm, we found that microglia are associated with formation of fibrovascular membranes and can promote pathological neovascularization. GO and KEGG results showed that PI3K-AKT pathway activation in retinal microglia was associated with pathological neovascularization, and PIK3IP1 was associated with retinal microglia activation. Then we used PCR, Western blot and Elisa techniques to confirm that the expression of VEGFA, FGF2, HGFα and MMP9 was increased in microglia after Lipopolysaccharide (LPS) induction. We also used cell flow cytometry and OIR models to verify the role of PI3K-AKT pathway and PIK3IP1 in microglia. Targeting of PIK3IP1 regulated the activation of the PI3K-AKT pathway in microglia, microglia function activation, and pro-angiogenic effects. These findings reveal the role of M1-type microglia in pathological neovascularization and suggests that targeting the PI3K-AKT pathway in microglia may be a new strategy for treating retinal pathological neovascularization.

## Introduction

Diabetic retinopathy is one of the major causes of vision loss in diabetic patients, especially when the disease progresses to proliferative diabetic retinopathy, where the appearance of fibrovascular membranes can lead to vitreous cavity hemorrhage and retinal detachment by retinal traction, leading to blindness in patients^1,2^. Pathological neovascularization is the main pathological feature of proliferative diabetic retinopathy^3,4^. Studies have focused on inhibition of endothelial cell proliferation and migration, as well as on pericyte protection and recruitment^5^. However, there are photoreceptor cells, ganglion cells, glial cells, and rpe cells in the retina. Apart from endothelial cells and pericytes, diabetes disrupts the functions of almost all retinal cells^3,6^. Even though the roles of other cells within the retina in pathological neovascularization have been assessed, the effects and specific mechanisms of various cells on pathological neovascularization have yet to be established^7,8^. Proliferative vitreoretinal disease (PVR) refers to pore-derived retinal detachment (RRD) and the extensive proliferation of intraocular cells in the retina and vitreous after surgery to form a fibroproliferative membrane, followed by fibroproliferative membrane contraction and retinal pulling. The fibroproliferative membrane that forms lacks neovascularization, thus providing a contrast to the fibrovascular membrane (FVM)^9^.

Retinal microglia, the tissue-specific macrophages that reside in the retina, originate from yolk sac progenitor cells with high plasticity^10^. They can be polarized by sensing various stimuli in the local retinal microenvironment, and polarized microglia can perform immune defense functions^7,11,12^. In variance analysis after performing gene sequencing on microglia from normal individuals and PDR patients, there was a high degree of similarity between differential genes and known disease-related genes, suggesting that microglia are the primary bearers of disease occurrence and development^13,14^. Microglia are also involved in disruption of the blood-retinal barrier in the early stages of diabetic retinopathy and pathological neovascularization is inhibited after specific removal of retinal microglia^15–18^. Moreover, M1 and M2 phenotypic conversion as well as self-stabilization of microglia are important in pathological neovascularization^19–21^. The above studies imply that microglia are involved in pathological retinal neovascularization^6,15,22^.

The fibrovascular membrane (FVM) is an important marker for PDR as well as a good clinical sample from patients with diabetic retinopathy. Fibrovascular membranes have been sequenced, but only at the tissue level. Cellular composition and interactions of FVM have yet to be established^23^. Recently, investigators performed single-cell sequencing techniques on fibrovascular membranes and found that microglia are the most abundant cells, confirming pro-fibrotic effects of microglia^9^. The microglia can also promote pathological neovascularization; however, the exact mechanisms remain unclear. Oxygen-induced retinopathy (OIR) animal models are commonly used in ophthalmology to study proliferative diabetic retinopathy^20,24,25^. Therefore, we investigated the specific mechanisms and pathways by which microglia promote neovascularization.

## Results

### Scissor algorithm revealed that microglia promotes the formation of fibrovascular membranes

**Figure 1:**
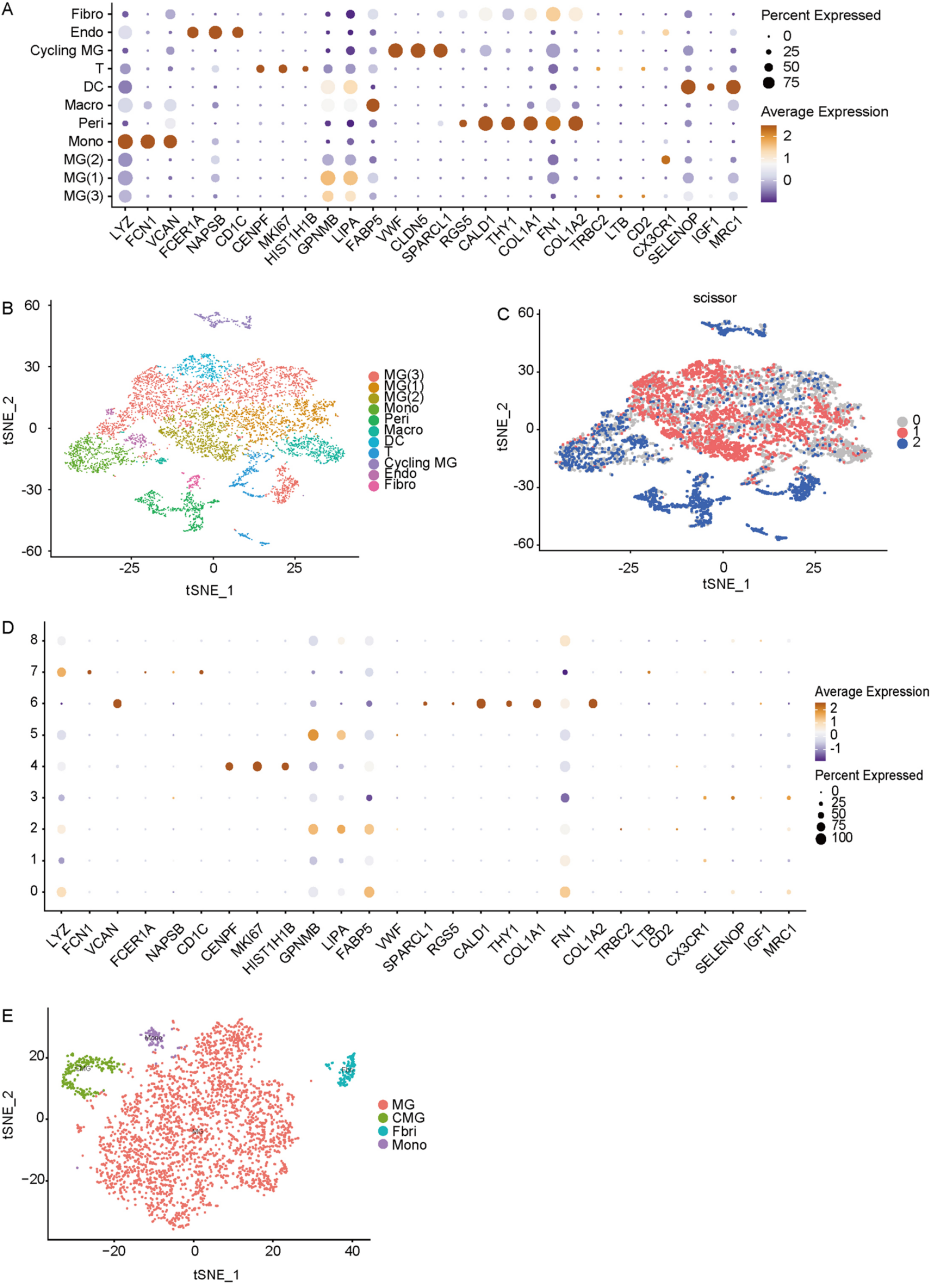
Single-cell RNA-seq identified microglia as the major cell population in the retinal fibrous vascular membrane and revealed that microglia were positively related to the progression of PDR. (**A**) The characteristic genes showed that there were 8 kinds of cells in the fibrous vascular membrane, namely microglia (MG), monocyte (Mono), macrophage (Macro), fibroblast (Fibro), pericyte (Peri), endothelium (Endo), dendrite cells (DC) and T lymphocyte (T). (**B**) t-SNE (t-Distributed Stochastic Neighbor Embedding) dimension reduction plot showed that a total of 20,913 genes and 7581 cells were obtained, of which microglia accounted for the majority. (**C**) Scissor showed that the Scissor^+^ cells were mainly concentrated in the microglia, after setting alpha as 0.5 and family as “binary” for calculation. (**D,E**) The characteristic genes showed that there are 3 kinds of cells in the fibro-vascular membrane, namely microglia (MG), monocyte (Mono), and fibroblast (Fibro), of which microglia accounted for the majority.

To establish which cellular components are associated with formation of PDR fibrovascular membranes, we downloaded the GSE165784 sequencing data from the GEO database. Then, we normalized the expression matrix in Rstudio using the Seurat R package (version 4.1.0), and performed clustering analysis according to the genes shown in Fig. 1A to obtain a total of 20,913 genes and 7,581 cells (MG(3): 2507; MG(2): 898; MG(1): 1011; Mono: 645; Peri: 590; Macro: 549; DC: 400; T: 412; Cycling MG: 274; Endo: 174; Fibro: 121), with microglia being the most abundant (Fig. 1B).

The Scissor R package was used to quantify similarities between each cell and each ontology sample to integrate the phenotypically relevant bulk expression data and single-cell data^26^. Then, the Scissor R package used the sample phenotypes to optimize regression models on the correlation matrix to identify relevant subpopulations, helping us identify cell populations that were most relevant to the phenotype. The bulk sequencing information was divided into two groups, one for 20 normal and 51 NPDR and one for 5 PDR. Then, we imported the phenotype information with alpha as 0.5 and family as binomial for calculation (Fig. 1C). Red represents Scissor^+^ while blue represents Scissor^-^. Red is mainly concentrated in microglial fractions, indicating that microglia may be involved in formation of fibrovascular membranes and play a positive role in promoting it. Moreover, we performed a clustering analysis of the PVR information in GSE165784 and found that microglia were highly abundant in fibrovascular membranes (Fig. 1D and E).

### Activation of the PI3K-AKT pathway in microglia may be involved in pathological neovascularization in fibrovascular membranes

**Figure 2:**
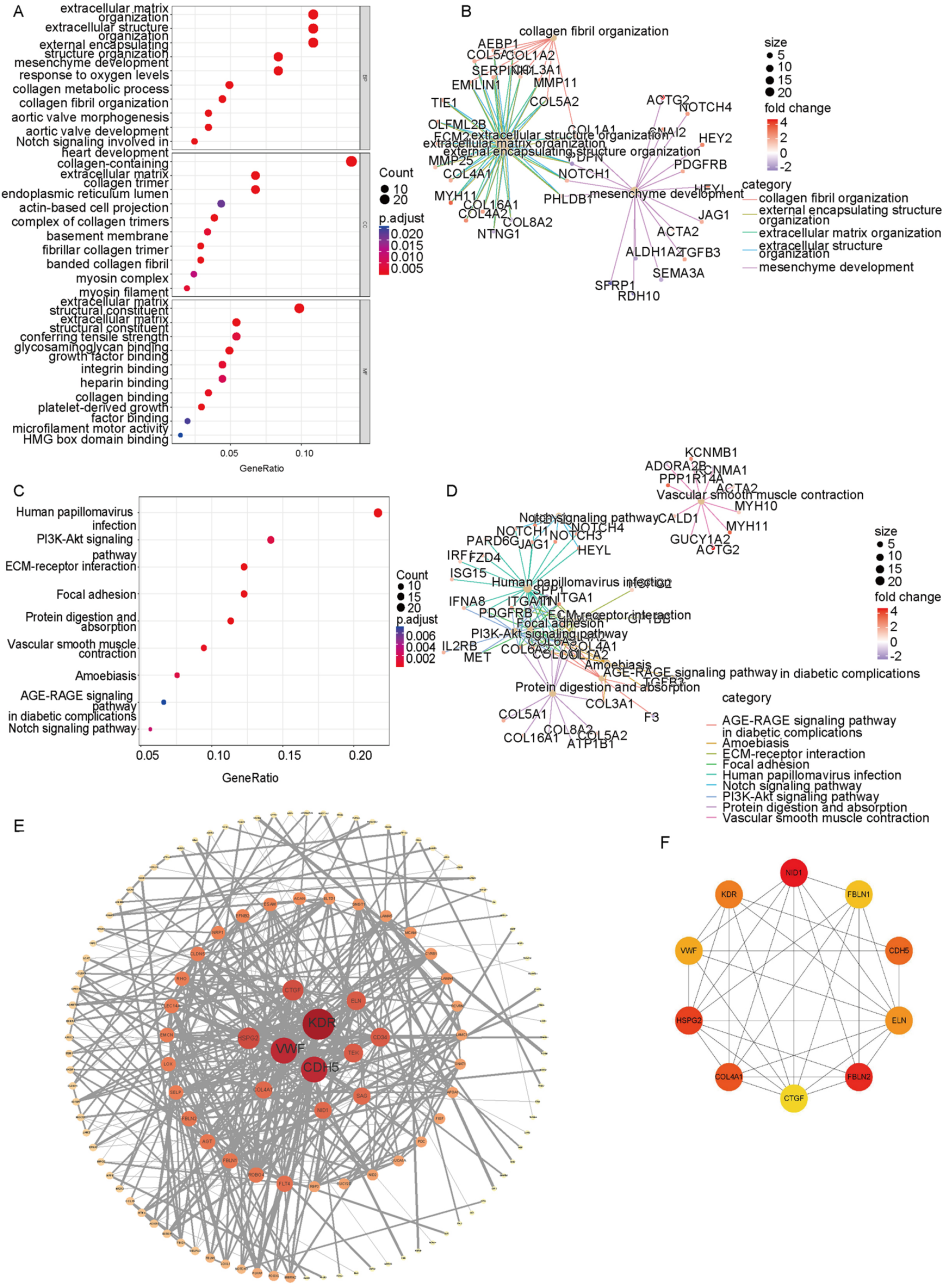
Bulk RNA-seq revealed that PDR exhibited more pronounced pro-angiogenic activity in all cell clusters in the FVM. (**A,B**) GO enrichment analysis showed that the fibrovascular membrane exhibited stronger pro-angiogenic activity. (**C,D**) KEGG enrichment analysis showed that several pathways enriched were involved in angiogenesis, including the PI3K-Akt pathway. (**E,F**) Protein–Protein Interaction (PPI) network prediction showed the differentially expressed genes that are most actively involved in protein interactions.

To establish the biological functions and possible active signaling pathways of fibrovascular membranes, we selected fibrovascular membranes in PDR and fibroblasts in PVR for exploration. We downloaded two datasets (GSE41019 and GSE60436) with information on three PVR fibrovascular membrane samples and six PDR fibrovascular membrane samples, respectively, and uploaded them to Network Analyst for analysis to obtain a list of differentially expressed genes. We screened the down-regulated and up-regulated genes according to adj. *P*. Val < 0.05, logFC < − 1, and logFC > 1, respectively. Then, we performed the GO and KEGG pathway enrichment analyses using “clusterProfiler” in R. GO enrichment analysis revealed that the differentially expressed genes were mainly concentrated in extracellular matrix formation, angiogenesis, and intercellular adhesion, while KEGG results showed that the pathways were mainly enriched in PI3K-Akt, extracellular matrix production, intercellular tight junctions, angiogenesis, and the Notch signaling pathway (Fig. 2A–D). The PI3K-AKT pathway is an intracellular signaling pathway that responds to extracellular signals to promote metabolism, proliferation, cell survival, growth, and angiogenesis^27^. These results suggest that the PI3K-AKT pathway is one of the major active signaling pathways in fibrovascular membranes. We also uploaded the list of differentially expressed genes to the string database for protein interaction prediction. It was found that KDR, VWF, CDH5, and CTGF were the four highest-scoring genes (Fig. 2E and F). Among them, KDR, VWF, and CDH5 are highly correlated with angiogenesis, while CTGF is associated with fibrosis^28–31^. These above findings show that angiogenesis and fibrosis are the main pathological features of PDR.

**Figure 3:**
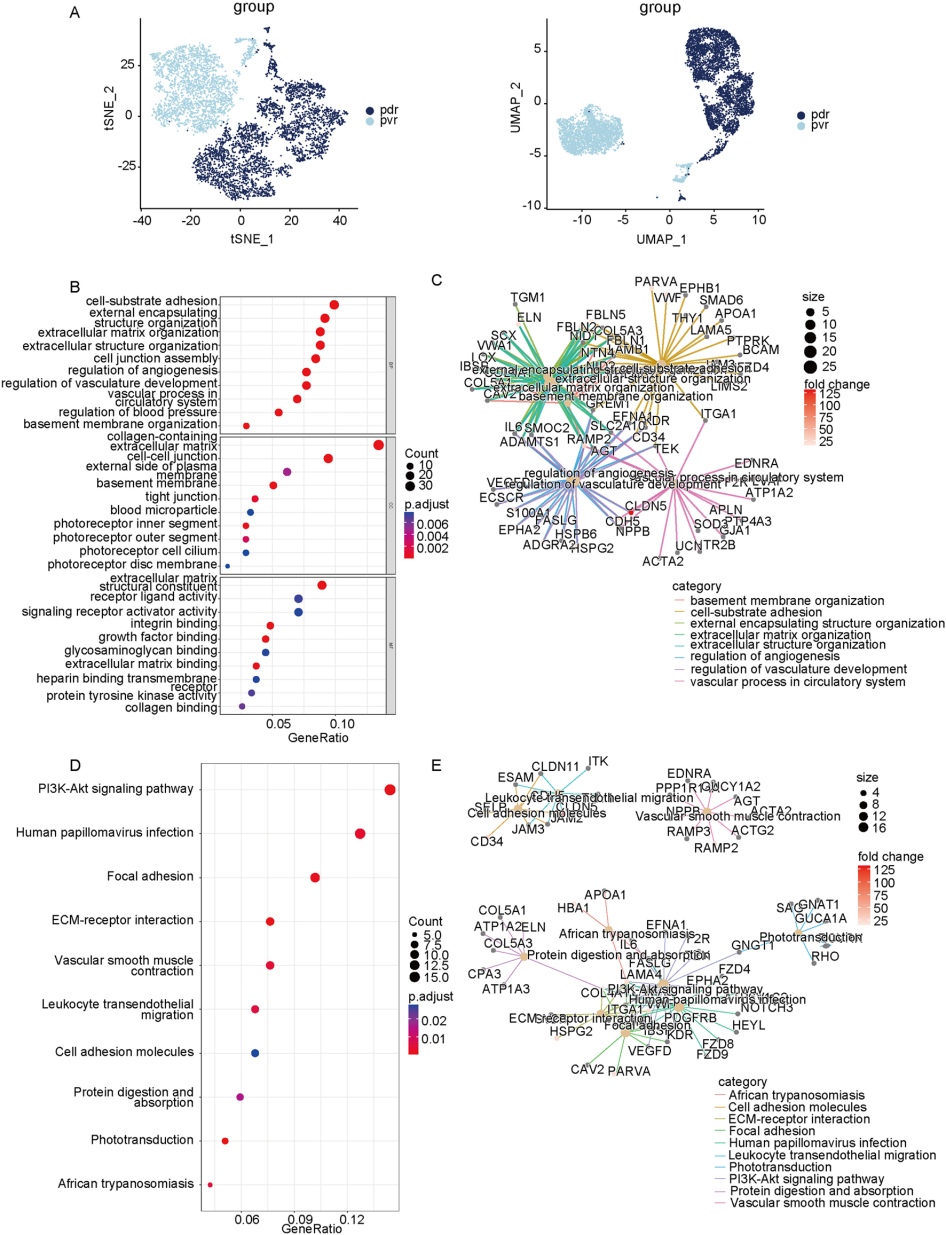
Enrichment analysis showed that microglia in the PDR membrane exhibited stronger pro-angiogenesis activity. (**A**) t-SNE and UMAP plots showed that PDR microglia and PVR microglia were successfully combined. (**B,C**) GO enrichment analysis showed that the genes upregulated in the PDR microglia were predominately involved in angiogenesis. (**D,E**) KEGG enrichment analysis showed that the genes upregulated in the PDR microglia were predominately involved in angiogenic pathways.

To establish the biological functions and specific active signaling pathways of microglia in fibrovascular membranes, we extracted microglia in PDR and PVR using the subset function and integrated them (Fig. 3A). Then, differential analysis was performed using the DEsingle function after which GO and KEGG analyses were conducted. GO enrichment analysis showed the extracellular matrix formation, intercellular adhesion, and angiogenesis were enriched while KEGG results showed that the PI3K-Akt signaling pathway, human papillomavirus infection, focal adhesion, and ECM-receptor interaction were enriched (Fig. 3B–E). Combined with findings from tissue sequencing, findings from differential analysis suggests that PI3K-AKT pathway activation in microglia may be one of the important reasons for fibrovascular membrane formation.

### PIK3IP1 expressions were suppressed in pathological retinal neovascularization

**Figure 4:**
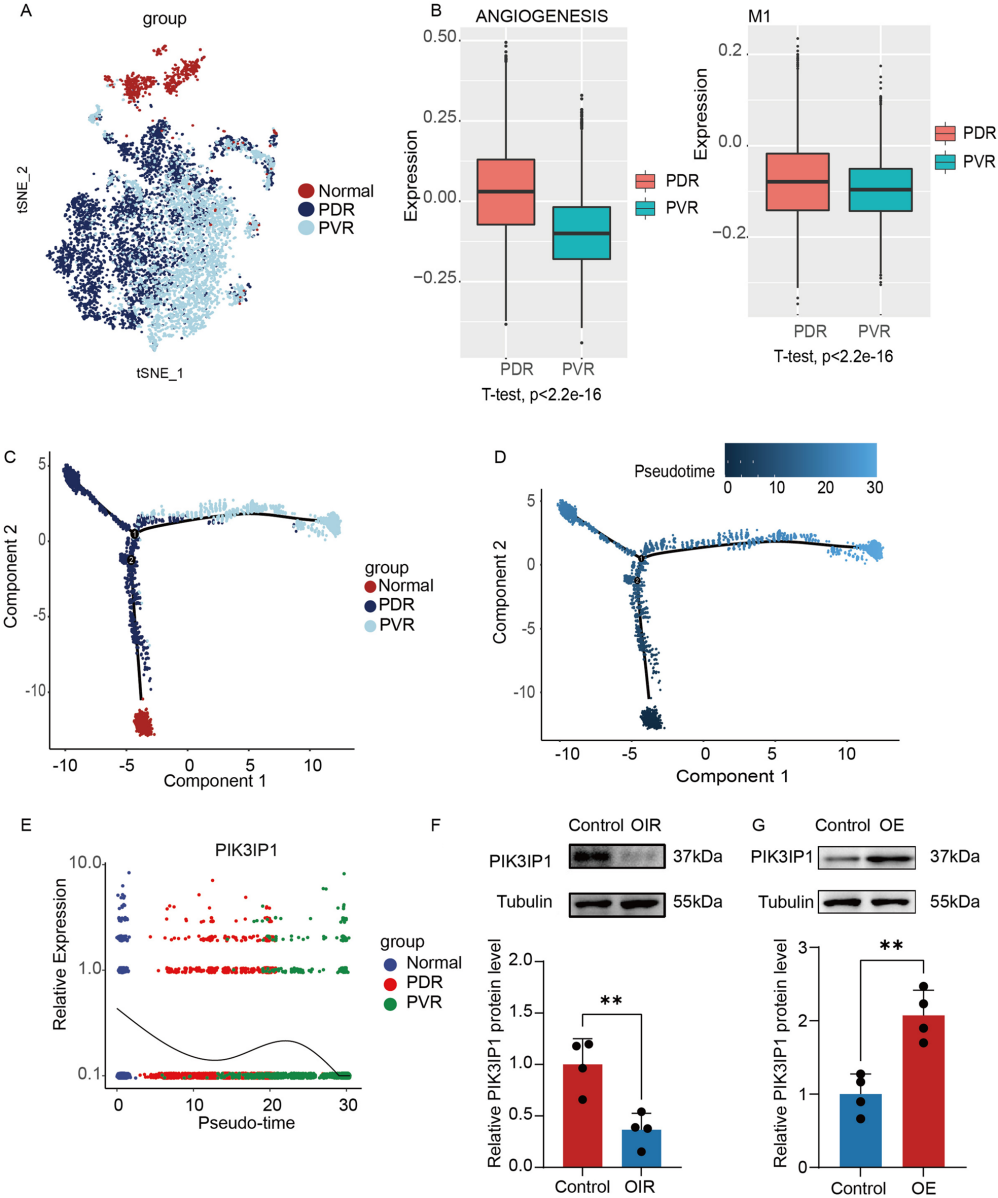
The expressions of PIK3IP1 were decreased in PDR microglia and in the retina of OIR mice model. (**A**) t-SNE plot showed that normal microglia, PDR microglia and PVR microglia were successfully integrated after using Harmony algorithm. (**B**) GSVA analysis revealed that PDR microglia had more pronounced pro-angiogenic properties and M1 phenotype than PVR membranes. (**C–E**) Pseudo-time analysis showed that PDR and PVR microglia both originated from normal microglia and then differentiated in different directions. PIK3IP1 was downregulated in PDR microglia. (**F**) The results of Western blot showed that the expressions of PIK3IP1 in the retina of OIR mice were decreased. (n = 4). (*P < 0.05, **P < 0.01, ***P < 0.001). (**G**) Western blot analysis of PIK3IP1 level in PIK3IP1-overexpressed HMC3 cells transfected with control and plasmid (n = 4). (*P < 0.05, **P < 0.01, ***P < 0.001).

To investigate functional and typing differences between PDR and PVR microglia, we analyzed the microglia in angiogenic and macrophage differentiation pathways using the GSVA R package (version 1.40.1). The PDR microglia exhibited significant pro-angiogenic capacities compared with PVR microglia, while phenotypically tending towards the M1 pro-inflammatory phenotype (Fig. 4A).

To establish the associations and differences between PDR, PVR, and normal microglia, normal human microglia were downloaded from the GEO database and integrated with previous PDR and PVR microglia using the harmony function (Fig. 4B, Supplementary Fig. 1A–D). The monocle R package (version 2.20.0) was used to perform the pseudo time analysis. It was found that both PDR and PVR microglia differentiated from normal microglia, after which each differentiated in different directions (Fig. 4C,D). At the same time, PIK3IP1 expressions were markedly suppressed in PDR microglia (Fig. 4E), while changes in normal and PVR microglia were insignificant. PIK3IP1 is homologous to the PI3K regulatory subunit (p85), which interacts with the PI3K catalytic subunit (p110) and inhibits PI3K activities through its p85-like structural domain^32^. Suppressed expressions of PIK3IP1 may be one of the important reasons for activation of the PI3K-AKT pathway in PDR microglia.

**Figure 5:**
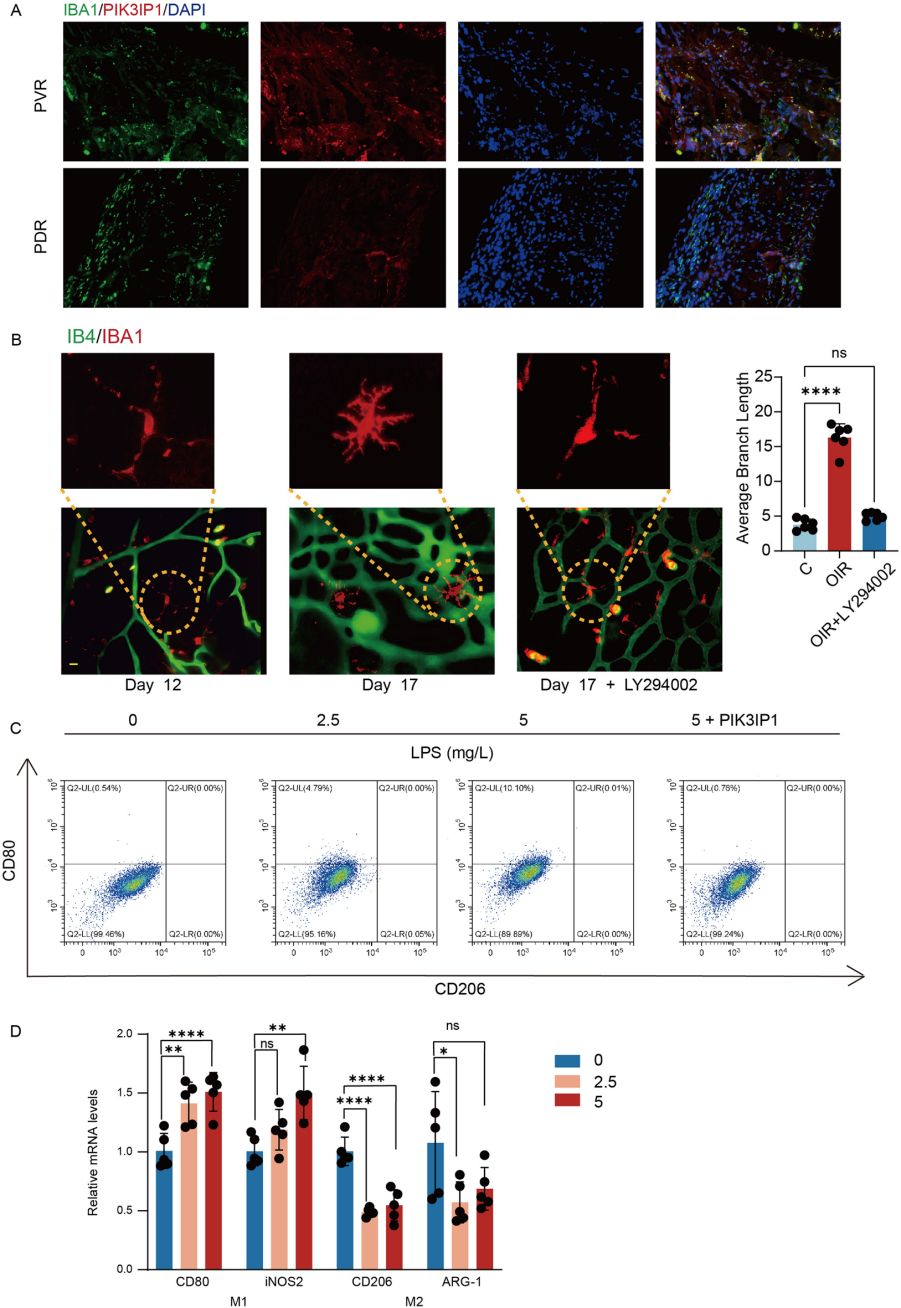
The PI3K-AKT pathway mediated microglial activation, and PIK3IP1 inhibited microglial differentiation towards the M1 phenotype. (**A**) The expressions of PIK3IP1 were reduced in fibrovascular membranes of PDR patients. (**B**) At 12, mice intravitreally injected with 1 μL PBS, 0.1% DMSO, LY294002(50 μM). LY294002 could inhibit the activation of retinal microglia in OIR mice. Scale bar, 50 μm (n = 6). (*P < 0.05, **P < 0.01, ***P < 0.001). (**C,D**) LPS induced the M1 phenotype of HMC3 cells. 24 h after administration with LPS, a significantly higher level of M1 marker expression was observed. Overexpression of PIK3IP1 could prevent the differentiation of HMC3 towards M1(n = 4). (*P < 0.05, **P < 0.01, ***P < 0.001).

The OIR model is a common animal model that mimics retinal pathological neovascularization. We collected the retinas of OIR mice and assessed PIK3IP1 expressions (Fig. 4F). On day 17, PIK3IP1 expressions were found to be suppressed in retinas of OIR mice, consistent with above prediction results. At the same time, we also detected the expressions of PIK3IP1 in the fibrovascular membrane of PVR patients and the fibrovascular membrane of PDR patients, and found that the expressions of PIK3IP1 were significantly reduced in the fibrovascular membrane (Fig. 5A). We then constructed a PIK3IP1-overexpressed HMC3 cell line, and the knockdown efficiency graph is shown in Fig. 4G.

### Activation of the PI3K-AKT pathway mediated microglia activation and pro-angiogenic capacities

To verify whether the PI3K-AKT pathway regulates microglial activation in vivo, we constructed OIR animal models, then collected the retinas of mice on days 12 and 17 for immunofluorescence staining. Isolectin B4 (IB4), a specific marker of microglia, can specifically bind vascular endothelial cells. On day 12, retinal microglia exhibited a branching resting state, and strongly IBA1-positive microglia appeared around the OIR retinal neovascularization on day 17, with the microglia being amoeboid and active with a significant increase in mean protrusion length. However, upon injection of LY294002 into the vitreous cavity of mice on day 12, changes in mean protrusion lengths of microglia were not significant (Fig. 5B). Therefore, the PI3K-AKT pathway is associated with microglial activation, in tandem with previous findings^24^.

**Figure 6:**
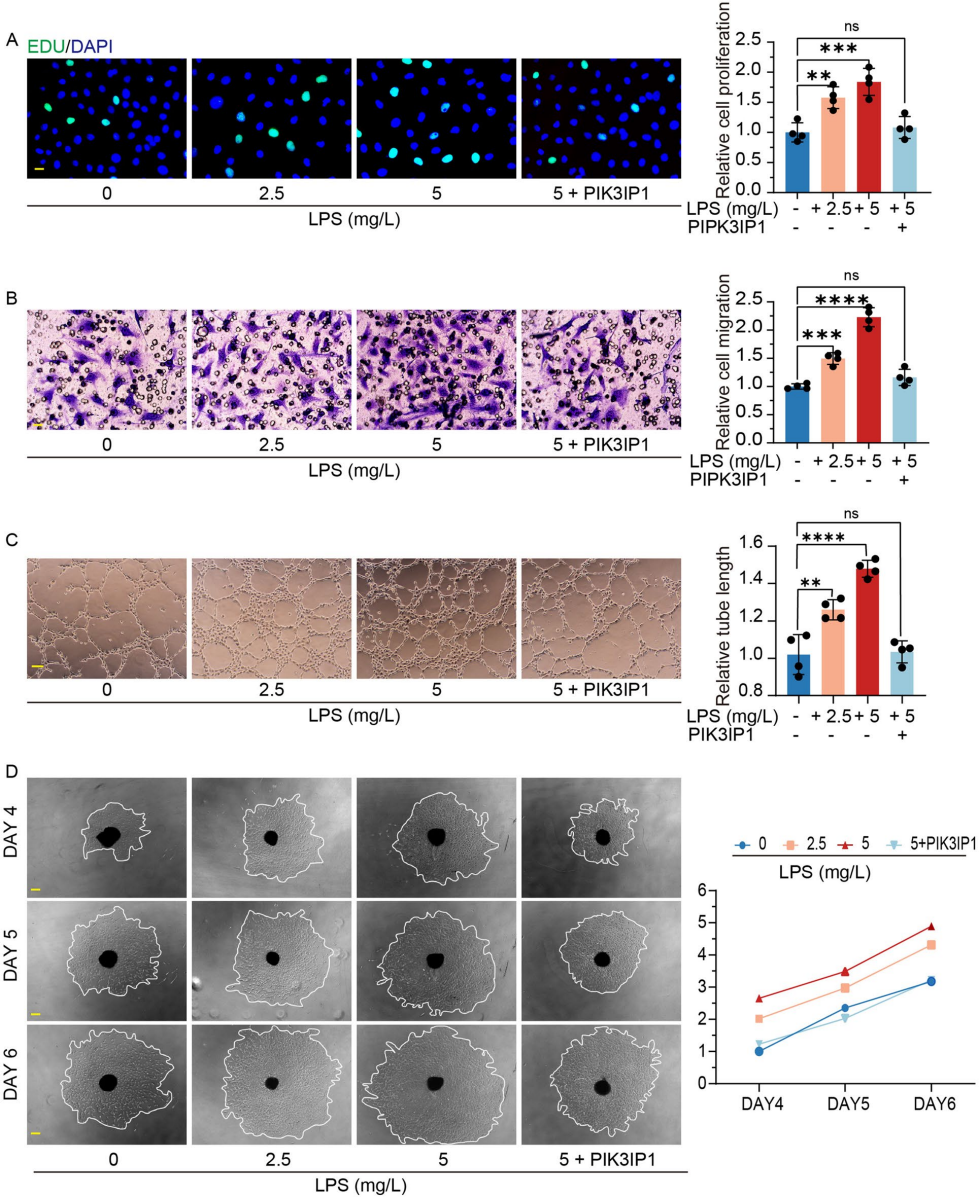
In *Vitro* experiments validated the angiogenic function of microglia. (**A–C**) Activated microglia enhanced HRVECs proliferation, tube formation, and migration. EdU assay showed that the proliferation rate of HRVECs increased significantly after co-culture. Scale bar, 20 μm (n = 4). Transwell experiment showed that the elevated migration capacity after co-culture. Scale bar, 20 μm (n = 4). Tube formation assay showed that the elevated tube formation capacity after co-culture. Scale bar, 100 μm (n = 4). (*P < 0.05, **P < 0.01, ***P < 0.001). (**D**) Sprouting was measured using an inverted microscope and expressed relative to control on day 4, day 5, and day 6. Scale bar, 200 µm (n = 4). (*P < 0.05, **P < 0.01, ***P < 0.001).

**Figure 7:**
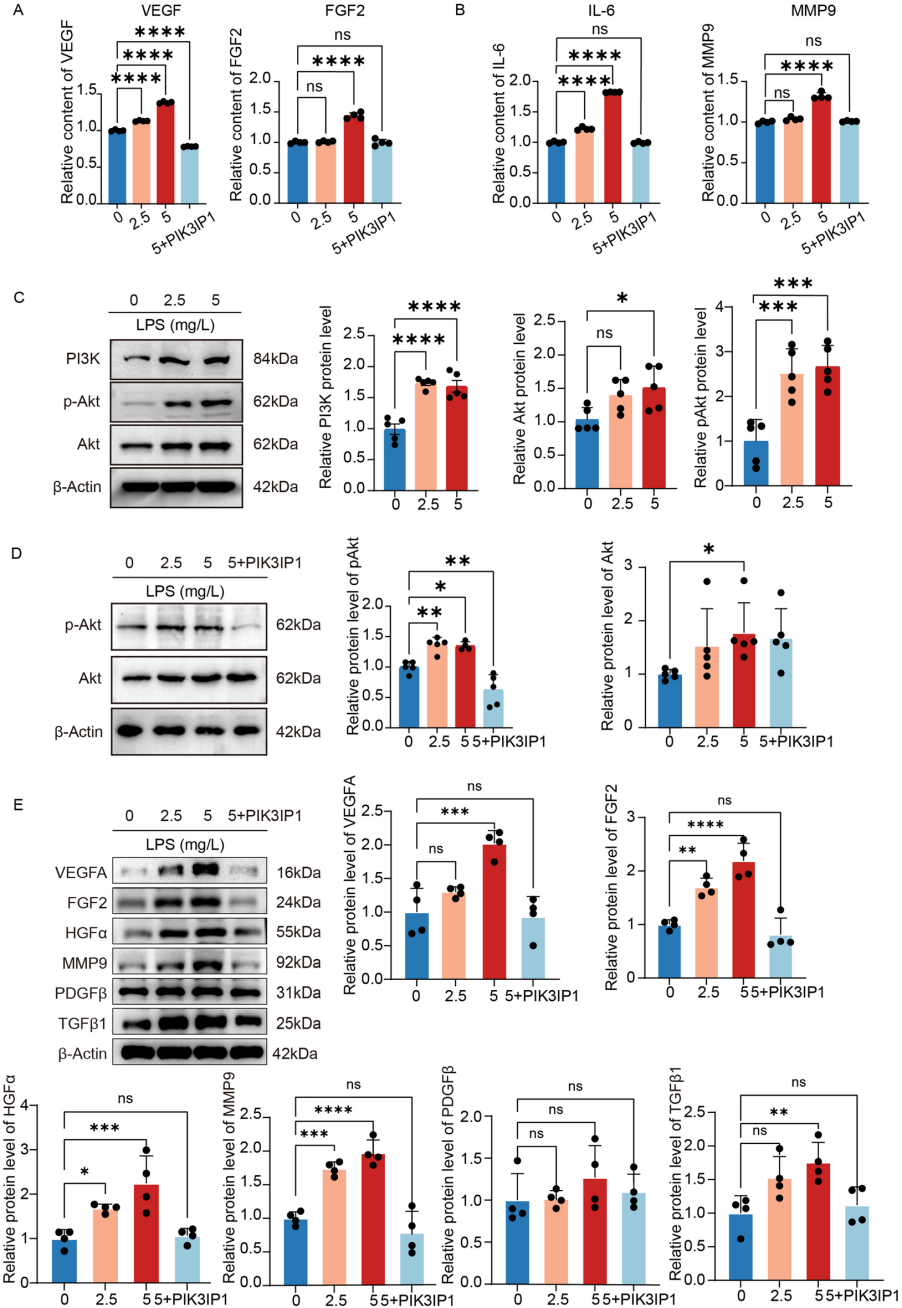
LPS promoted the release of growth factors and cytokines from microglia, and activated the PI3K-AKT pathway of microglia. (**A,B**) 24 h after administration with LPS, VEGF, FGF2, IL-6, and MMP9 were elevated in the supernatant of HMC3 cells (n = 4). (*P < 0.05, **P < 0.01, ***P < 0.001). (**C,D**) 24 h after administration with LPS, western blot analysis of PI3K, AKT and P-AKT level in HMC3 cells. ACTIN was detected as the loading control (n = 5). (*P < 0.05, **P < 0.01, ***P < 0.001). (**E**) 24 h after administration with LPS, western blot analysis of VEGFA, FGF2, HGFα, MMP9 and PDGFβ level in HMC3 cells. ACTIN was detected as the loading control (n = 4). (*P < 0.05, **P < 0.01, ***P < 0.001).

LPS-induced microglia activation is a common in vitro model of microglia inflammation and has also been applied in studies of pathological neovascularization in the eye^33^. We induced the activation of HMC3 cells with LPS for 24 h, and collected the supernatants as conditioned medium and co-cultured them with HR. After 24 h of induction, both HMC3 and BV2 differentiated towards the M1 phenotype, with elevated M1 marker expressions, while overexpressions of PIK3IP1 inhibited HMC3 and BV2 differentiation towards the M1 phenotype (Fig. 5C,D, Supplementary Fig. 1E), indicating that the conditioned medium for HMC3 significantly improved HR proliferation, tube-forming, and migration abilities, while BV2 conditioned medium promoted choroidal sprouting (Fig. 6A–D). Addition of the PI3K inhibitor (LY294002) to HMC3 induced the failure of the conditioned medium to improve HR proliferation, tube-formation and migration abilities (Supplementary Fig. 2A–C). In addition, we assessed the expressions of VEGF, ANG, TGFB1, HGF, PDGFB, and FGF2 in the microglia and found that HGF, PDGFB, and FGF2 expressions were elevated in the microglia, with the most significant increase being in FGF2 expressions, consistent with previous studies (Fig. 7E, Supplementary Fig. 2D). These results suggest that activation of the PI3K-Akt pathway in retinal microglia mediates microglial activation and pro-vascular activities.

### PIK3IP1 suppressed cytokine release from microglia by inhibiting PI3K-AKT pathway activation

We performed an ELISA test on the microglial medium after LPS stimulation and found that microglia activation was accompanied by increased expressions of cell growth factors (VEGF and FGF2), as well as increased levels of inflammatory factors (IL-6 and MMP9), while overexpressions of PIK3IP1 inhibited the increase in the above factors (Fig. 7A and B).

Then, we clarified whether PIK3IP1 can inhibit LPS-induced activation of the PI3K-AKT pathway in microglia. PI3K, AKT, and p-AKT all increased with increasing LPS concentrations (Fig. 7C). However, when PIK3IP1 was overexpressed, there was a significant decrease in AKT phosphorylation, with no significant effects on total AKT levels (Fig. 7D).

The above results suggest that PIK3IP1 is involved in pathological neovascularization by inhibiting PI3K-AKT pathway activation to suppress microglia differentiation to M1 type and release cytokines.

## Discussion

We analyzed the phenotypic and single-cell sequencing data in combination with the Scissor algorithm and found that retinal microglia in gene enrichment analysis were enriched in angiogenic regulation and extracellular matrix alterations, PI3K-Akt activation, and PIK3IP1 downregulation in PDR microglia. Then, we verified that microglia can be activated to M1 type upon induction by LPS inflammatory stimuli and that activation of the PI3K-Akt pathway is accompanied by upregulation of expressions and release of relevant pro-angiogenic factors in the microglia. Therefore, microglia in the inflammatory environment has significant pro-angiogenic effects, and both microglia activation and pro-angiogenic activities were inhibited after overexpression of PIK3IP1.

In PDR, elevated levels of vascular growth factors and inflammatory factors led to enhanced proliferation, migration, tube formation, and ultimately, neointima formation in retinal endothelial cells^2^. We extracted supernatants from LPS-stimulated microglia and found that the supernatants promoted HR proliferation, migration, and tube-forming abilities. Treatment of microglia with the PI3K inhibitor (LY294002) and overexpression of PIK3IP1 inhibited the pro-angiogenic effects of LPS-stimulated microglia. These results suggest that the PI3K-Akt pathway can mediate the pro-angiogenic activities of microglia.

Microglia, which are derived from the mesoderm, play an immune surveillance role after tissue maturation^34^. They are the main sources of inflammatory factors and can be activated to release cytokines when the internal environment is altered, mediating inflammatory responses and participating in repair processes of injured tissues^6,10,13,14^. Activated microglia can be divided into two types: M1 and M2 phenotypes^12^. The M1 phenotype microglia are amoeboid and release various pro-inflammatory factors to induce inflammatory responses^35^. The M2 phenotype microglia are branched and secrete anti-inflammatory factors to inhibit inflammatory responses^12^. Studies have reported that the most prominent macrophage population in retinal angiogenesis are M2 macrophages, however, we found that M1 phenotype microglia also have significant pro-angiogenic effects^19^. The M1 phenotype of microglia can up-regulate the expressions of several pro-angiogenic factors, while FGF2 expressions are significantly increased. Physiologically, VEGF is a major trigger of pathological neovascularization, while FGF2 is an important pathway via which the microglia regulates neovascularization and fibrosis^25,36–39^. PI3K-AKT plays an important role in regulating cell proliferation, differentiation, and apoptosis^40–42^. Hypoxia, inflammation, and high glucose stimulation can promote cellular production of VEGF via the PI3K-AKT pathway, which can in turn activate the PI3K-AKT pathway, creating a vicious cycle^43–45^. Targeting the PI3K-AKT pathway to inhibit neovascularization is a promising therapeutic strategy^43^. We found that microglia were activated towards the M1 phenotype and that upregulation of pro-angiogenic factor expressions might be due to activation of the PI3K-AKT pathway. PIK3IP1, whose structure is similar to that of the PI3Kp85 subunit, was previously identified as a negative regulator of T cell activation to competitively inhibit PI3K-AKT pathway activation^32^. Our study showed that PIK3IP1 inhibited PI3K-AKT pathway activation in the microglia and the pro-angiogenic effects of microglia.

This study has some limitations. Due to limitations of the sample source, not all cells in the retina were included in the single-cell sequencing results. In contrast, only some cells of the retina such as microglia, endothelial cells, and pericytes were included in our study, thus, the roles of other cells of the retina in diabetic retinopathy and pathological neovascularization could not be explored. In addition, we only assessed the pro-angiogenic effects and mechanisms of M1 phenotype microglia in proliferative diabetic retinopathy, thus, the effects and mechanisms of M2 phenotype microglia in pathological neovascularization should be investigated further.

In conclusion, the PI3K-AKT pathway is involved in regulation of microglial activation and plays pro-angiogenic roles. We also found that targeting PIK3IP1 can specifically inhibit microglial activation towards the M1 phenotype, which is a potential strategy for treatment of diabetic retinopathy.

## Materials and methods

### Single-cell data processing and scissor identification

The PDR data in GSE165784 was downloaded from the GEO database and the expression matrix was normalized in R studio using R package *Seurat* (version 4.1.0). Downloaded from the GEO database, retinal Bulk sequencing data included 20 normal, 51 NPDRs, and 5 PDRs. We subsequently divided the retinal bulk sequencing information into two groups (20 normal and 51 NPDRs as a group, 5 PDRs as a group), using the R package *Scissor* (version 2.0.0)^26^. Phenotypic information, bulk sequencing information, and single-cell sequencing information were used for disease correlation analysis. The Scissor^+^ cell population was indicated to be positively associated with disease, the Scissor^-^ cell population was negatively associated with disease, and microglia were found to be mainly associated with the occurrence and development of fibrous vascular membranes. At the same time, the PVR expression matrix in GSE165784 is normalized.

### Bulk sequencing differentially expressed gene analysis

GSE41019 and GSE60436 datasets were downloaded from the GEO database (https://www.ncbi.nlm.nih.gov/geo/). Network Analyst (https://www.networkanalyst.ca) was used for RNA-seq data analysis, and eventually, we obtained a list of differentially expressed genes (DEGs)^46^. The threshold used for DEG screening was adjusted *P* value < 0.05 and |logFC|> 1. The volcano diagram was drawn using the ggplot2 R package (version 3.3.5) and the Gene Ontology (GO) enrichment analysis and Kyoto Encyclopedia for Genes and Genomes (KEGG) pathway enrichment analysis were performed using the R package *ClusterProfiler* (version 4.0.5) with the DEGs we previously obtained.

### Protein–protein interaction network

Using the STRING database, a protein–protein interaction (PPI) network to explore the interaction between the regulated proteins. Cytoscape software (version 3.3.0) and *CytoHubba* was also used in constructing and analyzing the network.

### Single-cell sequencing differential expressed gene analysis

The microglia subpopulation was extracted from GSE165784, the differential analysis was performed using R package *DEsingle* (version 1.12.0), the upregulated genes were selected and the GO and KEGG enrichment analysis was performed with R package *ClusterProfiler*.

### Pseudotime analysis

The normal human retinal single cell data GSE148077 dataset was downloaded from the GEO database, and the normal microglial population was extracted, the R package *Harmony* (version 0.1.0) was integrated with PDR and PVR single-cell sequencing data, and the R package *monocle* (version 2.20.0) was used for pseudotime analysis.

### GSVA analysis

Gene Set Variation Analysis (GSVA) is a particular type of gene set enrichment method that works on single samples/cells and enables pathway-centric analyses of sequencing data by “performing a conceptually simple but powerful change in the functional unit of analysis, from genes to gene sets^47^. Each cell was scored using the GSVA R package (version 1.40.1). The datasets related to angiogenesis, energy metabolism, and cell death were downloaded from MsigDB (www.gsea-msigdb.org) and plotted using the ggplot2 R package (version 3.3.5).

### Cell culture

BV-2 cells and Human Retinal Vascular Endothelial Cells (HRVEC) cells were purchased from the cell bank of the National Academy of Science (Shanghai, China). Cells were cultured in Dulbecco’s modified Eagle medium (DMEM, Gibco, Shanghai, China) supplemented with 10% FBS (AusgeneX, Gold Coast, Australia).

### Preparation of HCM3-CM and BV2-CM

HMC3 and BV2 were stimulated several concentrations (0, 2.5 and 5 ug/ml) of LPS for 24 h, the culture medium was aspirated, and the cells were rinsed thoroughly and cultured with fresh growth medium (MEM complete medium) for an additional 4 h. The CM was centrifuged, sterile filtered (0.22 µm filter), aliquoted and stored at − 80 °C. HMC3-CM and BV2-CM were used at equivalent dilutions in EC growth medium. Microglia-free medium with the same composition and conditions served as control medium.

### Animals

C57BL/6 mice were purchased from Nanjing Qinglongshan Experimental Animal Center (Nanjing, China) and kept in pathogen-free conditions with free access to food and water on a 12-h light/dark cycle. The mice were euthanized by 5% isoflurane inhalation. All animal treatments were approved by the Animal Ethics Committee of Nanjing Medical University, China, and performed in accordance with the Association for Research in Vision and Ophthalmology (ARVO) Statement for the Use of Animals in Ophthalmic and Vision Research. Experiments were conducted in accordance with the ARRIVE guidelines (https://arriveguidelines.org) and all methods were conducted in accordance with the relevant guidelines and regulations.

### Oxygen induced models

The OIR model was described previously^48^. Briefly, seven-day-old mouse pups along with their foster/nursing mothers were exposed to 75% oxygen for 5 days. At P12, the mice were returned to room air (RA, 21% oxygen), and the retinas were collected at P17. LY294002 was injected intravitreally into one eye of OIR mice on day 12 of age (day 5 after inducted with 75% oxygen) to inhibits PI3K-Akt pathway activation.

### IF staining

Eyes were enucleated and fixed in 4% paraformaldehyde for 2 h at room temperature. The intact retinas were collected, blocked and permeabilized in PBS containing 10% goat serum, 3% BSA, 1% Triton-X-100 and 0.2% Tween 20 for 1 h. Samples were then incubated with primary antibodies anti-rabbit IBa1 (1:300, ET1705-78, HuaBio), and isolectin B4 (1:50, L2895, Sigma) overnight at 4 °C, followed by incubation with fluorescence-conjugated cross-adsorbed secondary.

### Flow cytometry analysis

The cells were resuspended in FACS buffer and incubated in 100 µL FACS buffer containing 2 µL Fc block for 10 min at room temperature, and then stained with APC anti-mouse CD80 (1:50, E-AB-F0992E, Elabscience), and APC anti-human CD80 (1:50, E-AB-F1232E, Elabscience), and PE anti-human CD206(1:50, 321,105, Biolegend) for 30 min at room temperature. For staining intracellular antigens, after washing, cells were fixed and permeabilized using Methanol, and then stained with PE anti-mouse CD206 (1:50, E-AB-F1135D, Elabscience) and PE anti-human CD206(1:50, 321,105, Biolegend) for 30 min at room temperature. The cells were then washed with 1 mL FACS buffer, centrifuged at 500 *g* for 5 min at 4 °C, and resuspended in 0.2 mL FACS buffer and analyzed.

### qRT-PCR analysis and western blot

Total RNA of LPS activated and resting BV-2 cells were isolated using TrizolTM reagent (Life Technologies, CA, USA). Reverse transcription and qRT-PCR were conducted separately using a reverse transcription kit (Takara) and SYBR Green qPCR SuperMix kit (Invitrogen, Thermo-Fisher) according to the manufacturer’s protocol. The list of all primer pairs used for qRT-PCR is shown in Table 1 in Supplementary Material.

Western blot with transfected cells and retinas were conducted as described previously^48^. After centrifugation of cell lysates, protein was quantified with the BCA assay and then loaded onto an 8–13% SDS-PAGE gel. Primary antibodies used in this study were as follows: rabbit anti-PI3K p85 alpha (1:1000, ET1608-70, HUABIO), mouse anti-Pik3ip1 (1:1000, sc365777, Proteintech), rabbit anti-Akt (1:1000, 4685, Cell Signaling Technology)), rabbit anti-pAkt (1:1000, AF3262, Affinity), mouse anti-Beta tubulin (1:1000, EM1701-59, HUABIO), rabbit anti-VEGFA (1:1000, 50661S, Cell Signaling Technology), mouse anti-FGF2 (1:1000, SC-365106, Santa Cruz Biotechnology), mouse anti-HGFα (1:1000, SC-374422, Santa Cruz Biotechnology), mouse anti-MMP9 (1:1000, SC-13520, Santa Cruz Biotechnology), mouse anti-PDGFβ (1:1000, SC-365805, Santa Cruz Biotechnology), mouse anti-TGFβ1 (1:1000, SC-130348, Santa Cruz Biotechnology) and mouse anti-βactin (1:1000, M1210-2, HUABIO).

### Cell proliferation detected by EdU assay

To assess the proliferation viability of HRVECs, an EdU assay was carried out using BeyoClick™ EdU detection kits (Beyotime, Nanjing China). After the co-culture, HRVECs were incubated with EdU for 2 h, then fixed with 4% paraformaldehyde, blocked with 3% BSA, and permeabilized with 0.3% Triton X-100 for 15 min at room temperature. The cells were incubated with *Azide488* dye solution to label the proliferating cells. Cell nuclei were stained with DAPI and counted under a fluorescence microscope (Olympus, Tokyo, Japan).

### Cell migration assay

24 h after the co-culture, cells were seeded into the upper well for 12 h and allowed to invade through the transwell plate. The cells on the inserts were fixed with methanol, stained with crystal violet, and counted under a light microscope. The fold change of migrated cells through a transwell plate was recognized as the migration rate.

### Tube formation assay

The basement membrane matrix (Corning, NY, USA, 354,230) was placed into a 24-well plate and hardened at 37 °C for 30 min. HRVECs (1 × 10^5^ cells/well) were seeded on each well and incubated for 6 h at 37 °C. The images were taken under a light microscope.

### Choroidal sprouting assay

Retinal pigment epithelium (RPE)/choroid/sclera complex (also referred to as “choroid explant”) from 3-week-old wild-type (C57BL/6 J) male mice was dissected and cut into ~ 0.5 × 0.5 mm pieces. Choroid explants were then embedded into growth factor-reduced Matrigel (354,230, Corning, NY, USA) and cultured in DMEM supplemented with 10% FBS in a 24-well-plate. At days 3, 0.5 mL control medium or HMC3-conditioned medium was added per well. Endothelial sprouts were imaged using fluorescence microscope (Olympus, Tokyo, Japan). The sprouting area was quantified with Image J (NIH, Image J).

### Statistical analysis

All experimental data were illustrated as mean ± SD, and one-way ANOVA (analysis of variance) was conducted to calculate the significance of different sets of data. *P* < 0.05 was regarded as statistically significant.

### Supplementary Information

Supplementary Figure 1.

Supplementary Figure 2.

Supplementary Table 1.

Supplementary Information 1.

Supplementary Information 2.

## Data Availability

The datasets used and analyzed in this study are available from the corresponding author upon reasonable request. The datasets generated and/or analyzed during the current study are available in the GEO repository, https://www.ncbi.nlm.nih.gov/geo/.
