# Supplementary material for: Downregulation of PIK3IP1 in retinal microglia promotes retinal pathological neovascularization via PI3K-AKT pathway activation
Source: Sci Rep. 2023 Aug 7;13:12754. doi: 10.1038/s41598-023-39473-z (PMC10406944; doi:10.1038/s41598-023-39473-z)
Supplement: Supplementary file 1 — Supplementary Figure 1. [file 41598_2023_39473_MOESM1_ESM.pdf]

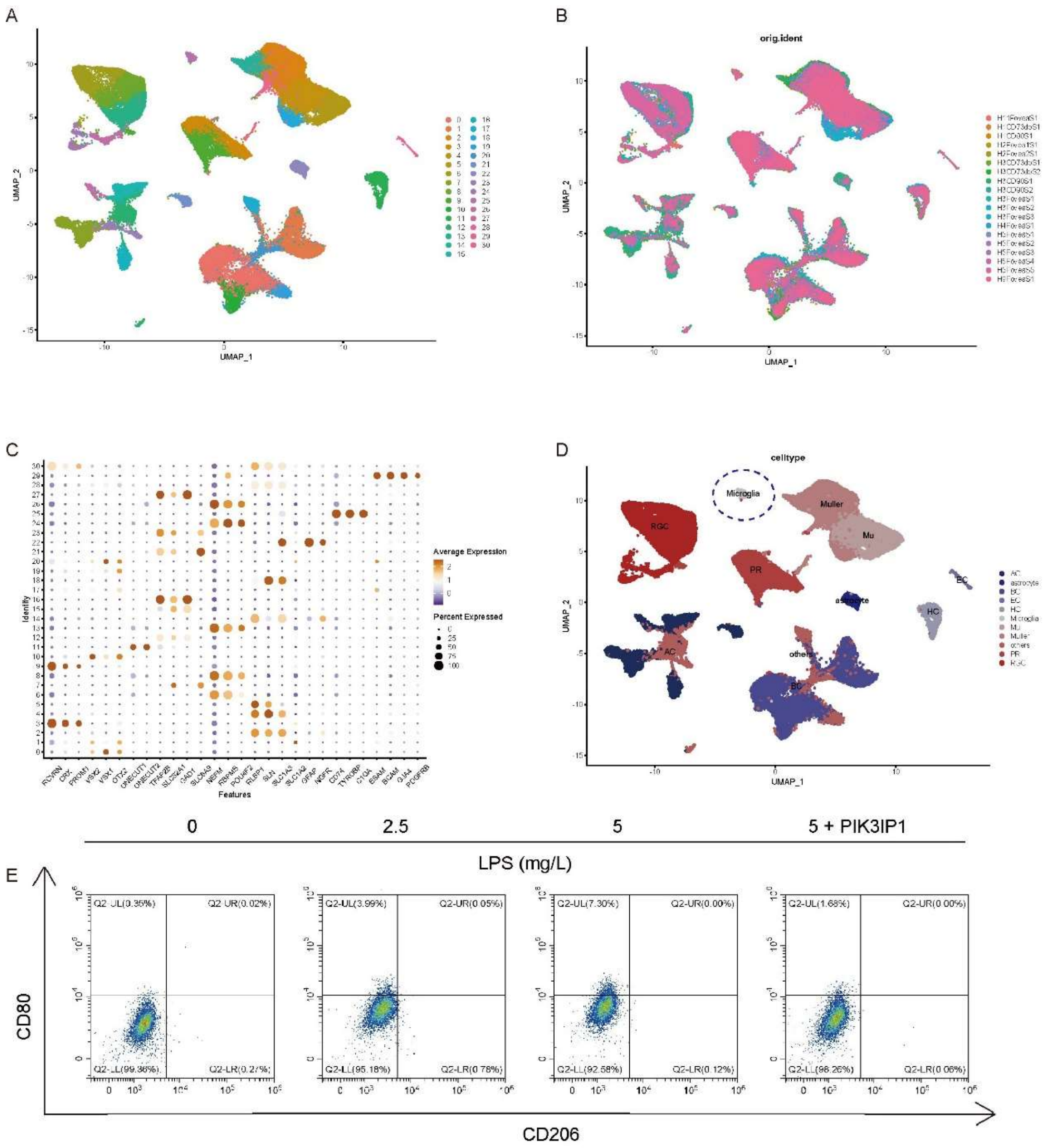

Supplementary Figure 1

Extraction of normal human retinal microglia from human foveal and peripheral retinal single-cell atlas (GSE148077), microglia overexpressed PIK3IP1.

(A) t-SNE dimension reduction plots of 86,253 human retinal cells. By setting the

resolution to 1.0, 31 clusters were identified.

(B) t-SNE dimension reduction plots of 86,253 human retinal cells after integration using *Harmony* algorithm. No significant batch effects were found after integration.

(C) The dot plot showed the expression marker genes expression within 31 clusters

(D) After identification of cell type, cells were annotated with different cell types on the t-SNE plot. Microglia were encircled in a dak blue circle.

(E) LPS induced the M1 phenotype of BV2 cells. (n=4). (**P*<0.05, ***P*<0.01, ****P*<0.001)
