# Supplementary material for: Downregulation of PIK3IP1 in retinal microglia promotes retinal pathological neovascularization via PI3K-AKT pathway activation
Source: Sci Rep. 2023 Aug 7;13:12754. doi: 10.1038/s41598-023-39473-z (PMC10406944; doi:10.1038/s41598-023-39473-z)
Supplement: Supplementary file 2 — Supplementary Figure 2. [file 41598_2023_39473_MOESM2_ESM.pdf]

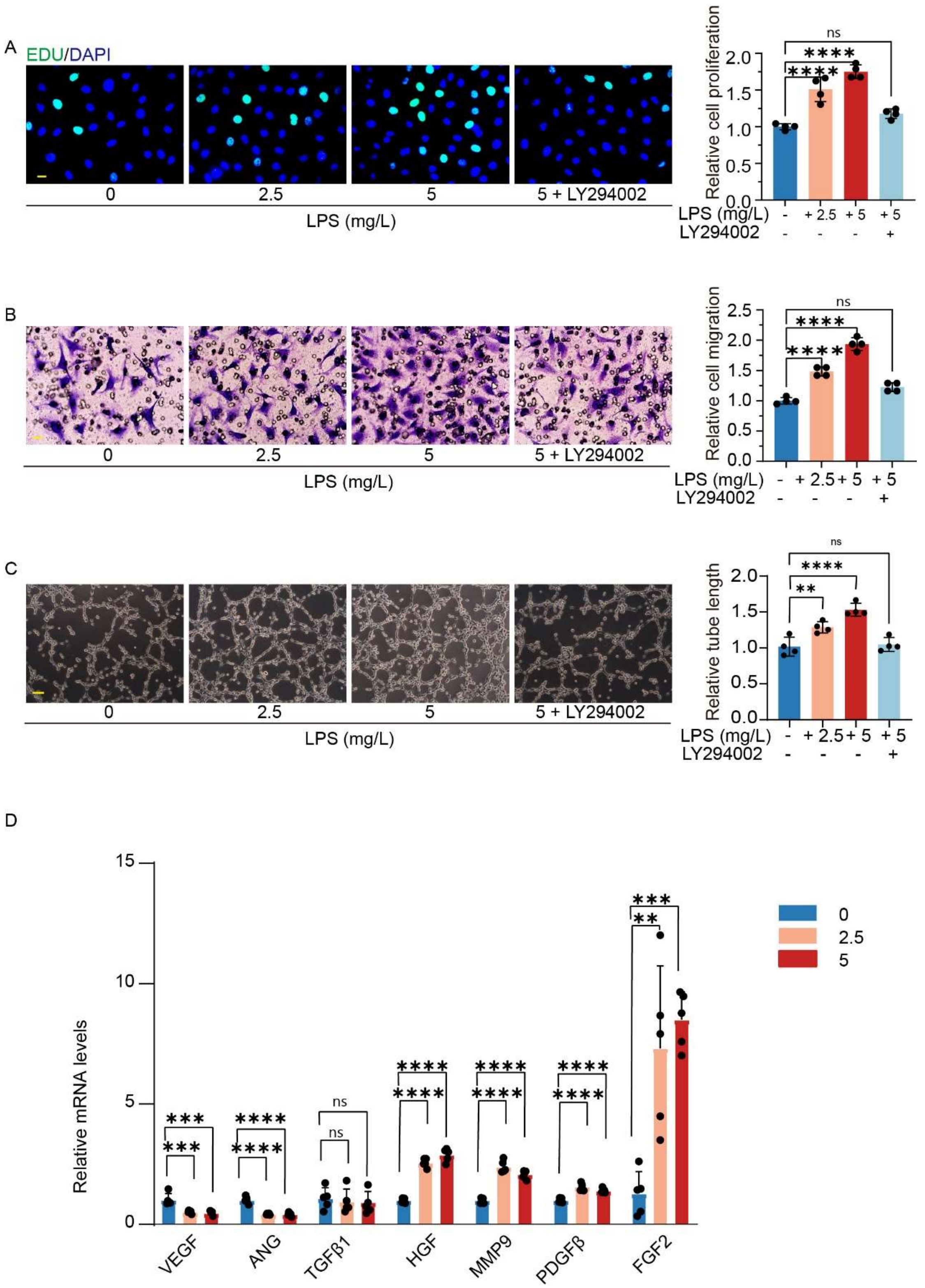

Supplementary Figure 2

LY294002 could inhibit the angiogenic function of microglia，and LPS promoted the expression of cytokines and growth factors in microglia.

(A, B, C) EdU assay showed that the proliferation rate of HRVECs increased

significantly after co-culture. Scale bar, 20 μm (n = 4). Transwell experiment showed that the elevated migration capacity after co-culture. Scale bar, 20 μm (n = 4). Tube formation assay showed that the elevated tube formation capacity after co-culture. Scale bar, 100 μm (n = 4). (*$P<0.05$, **$P<0.01$, ***$P<0.001$)

(D) 24 hours after administration with LPS, FGF2, MMP9 and HGF were upregulated in microglia. (*$P<0.05$, **$P<0.01$, ***$P<0.001$)
