# Supplementary material for: Downregulation of PIK3IP1 in retinal microglia promotes retinal pathological neovascularization via PI3K-AKT pathway activation
Source: Sci Rep. 2023 Aug 7;13:12754. doi: 10.1038/s41598-023-39473-z (PMC10406944; doi:10.1038/s41598-023-39473-z)
Supplement: Supplementary file 3 — Supplementary Table 1. [file 41598_2023_39473_MOESM3_ESM.pdf]

Supplementary Table 1

Target gene	Sequence
CD80.F	ACCCCCAACATAACTGAGTCT
CD80.R	TTCCAACCAAGAGAAGCGAGG
FGF2.F	GCGACCCACACGTCAAACTA
FGF2.R	TCCCTTGATAGACACAACTCCTC
iNOS2.F	CAGCTGGGCTGTACAAACCTT
iNOS2.R	CATTGGAAGTGAAGCGTTTCG
VEGF.F	TCACCAAAGCCAGCACATAGGAGA
VEGF.R	TTTCTCCGCTCTGAACAAGGCTCA
CD206.F	CTCTGTTCAGCTATTGGACGC
CD206.R	CGGAATTTCTGGGATTCAGCTTC
ARG1.F	CAGAAGAATGGAAGAGTCAG
ARG1.R	CAGATATGCAGGGAGTCACC
ACTB.F	GGCTGTATTCCCCTCCATCG
ACTB.R	CCAGTTGGTAACAATGCCATGT
MMP2.F	CCTGGACCCTGAAACCGTG
MMP2.R	TCCCCATCATGGATTCGAGAA
PGF.F	GTCTGCTGGGAACAACTCAACA
PGF.R	CACCTCATCAGGGTATTCATCCA
HGF.F	ATGTGGGGGACCAAACTTCTG
HGF.R	GGATGGCGACATGAAGCAG
MMP9.F	TTGAAGTCTCAGAAGGTGGAT
MMP9.R	GCAGGAGGTCGTAGGTCAC
PDGFB.F	CATCCGCTCCTTTGATGATCTT
PDGFB.R	GTGCTCGGGTCATGTTCAAGT
EGF.F	AGCATACTCAGCGAGAGAAGC
EGF.R	CCTTGTGAGAAAACTAGGAAGGG
TGFB1.F	ATGGTGGACCGCAACAAC
TGFB1.R	CCAAGGTAACGCCAGGAA
ANG.F	CCAGGCCCGTTGTTCTTGAT
ANG.R	GGAAGGGAGACTTGCTCATTC
