# Supplementary figures and images for: Downregulation of PIK3IP1 in retinal microglia promotes retinal pathological neovascularization via PI3K-AKT pathway activation
Source: Sci Rep. 2023 Aug 7;13:12754. doi: 10.1038/s41598-023-39473-z (PMC10406944; doi:10.1038/s41598-023-39473-z)

PI3K

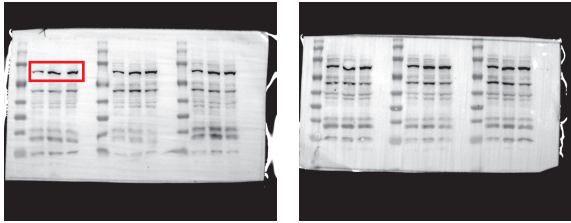

PI3K-ACTIN

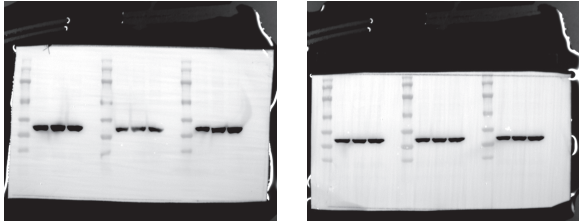

AKT

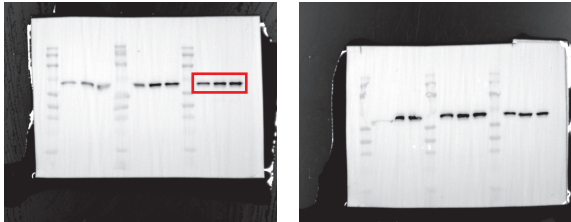

AKT-ACTIN

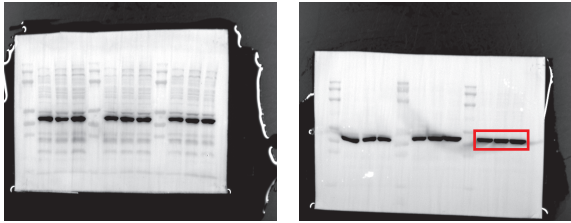

P-AKT

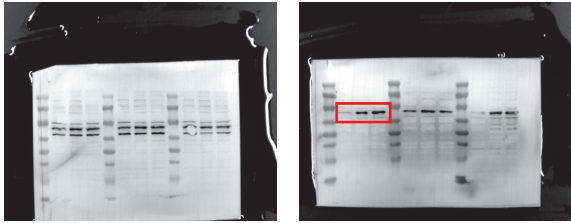

P-AKT-ACTIN

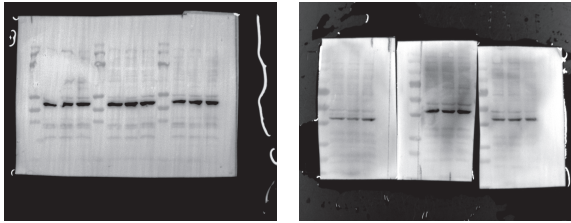

AKT

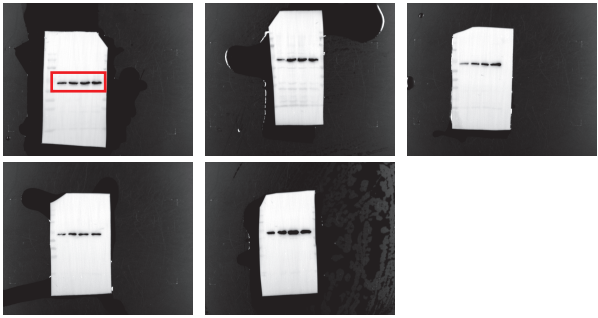

AKT-ACTIN

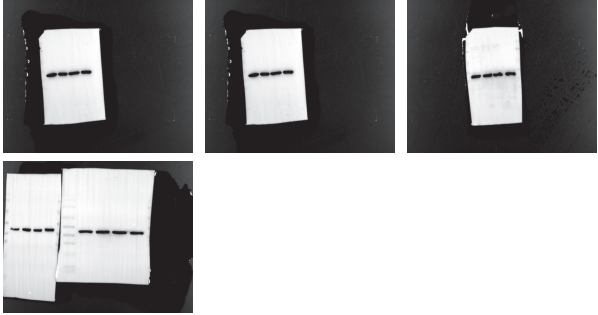

P-AKT

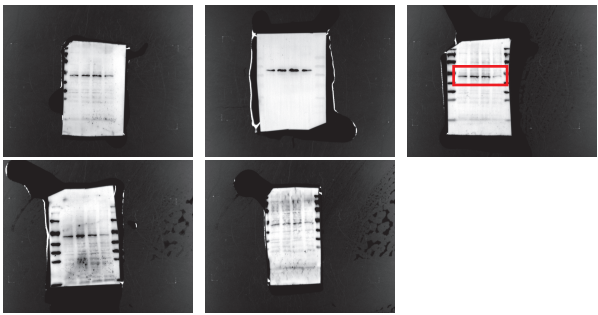

P-AKT-ACTIN

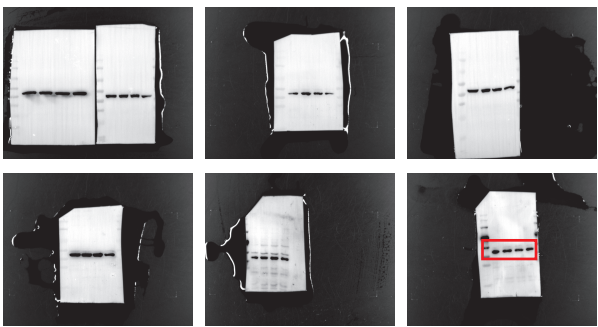

PIK3IP1

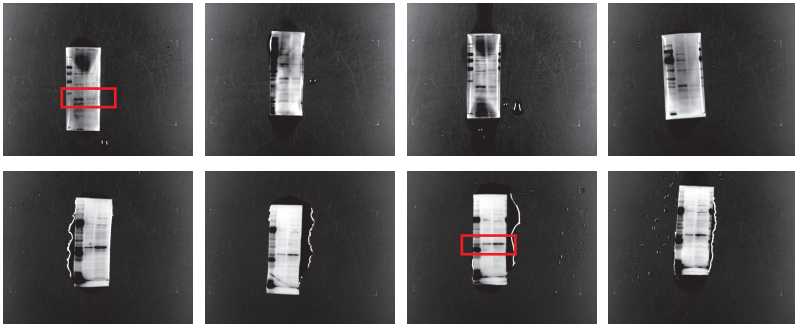

PIK3IP1 TUBULIN

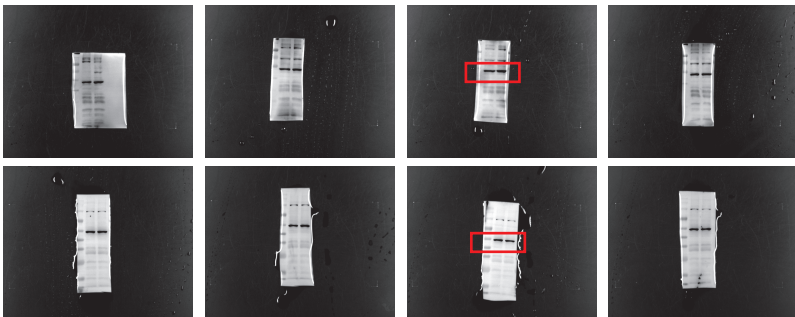

Supplement: Supplementary file 4 — Supplementary Information 1. [file 41598_2023_39473_MOESM4_ESM.pdf]

VEGFA

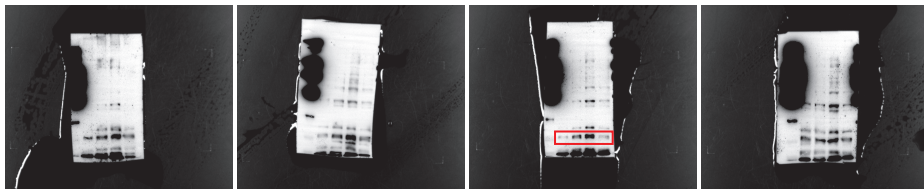

TGFβ1

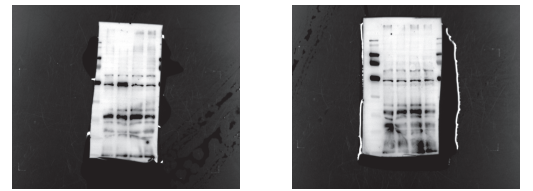

VEGFA-Actin

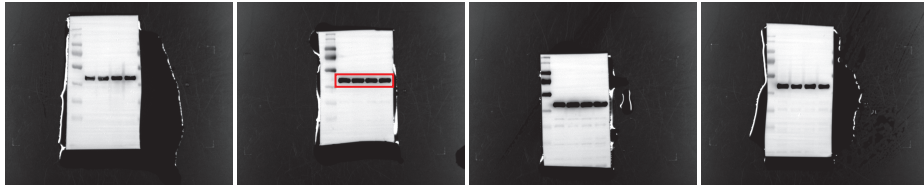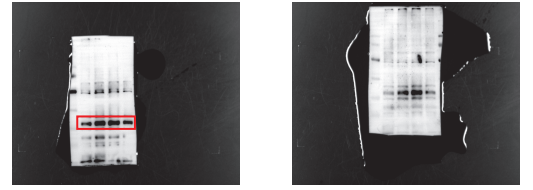

FGF2

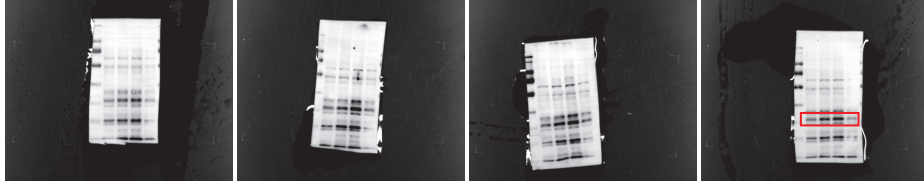

TGFβ1-Actin

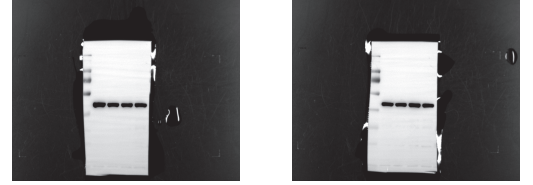

FGF2-Actin

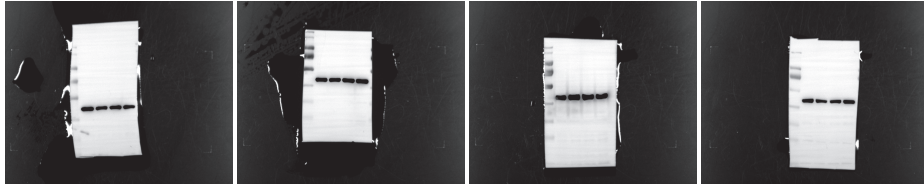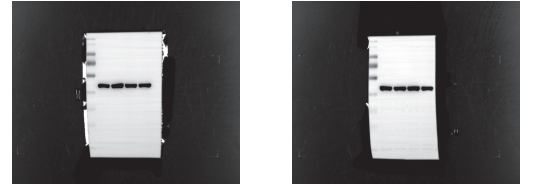

HGFα

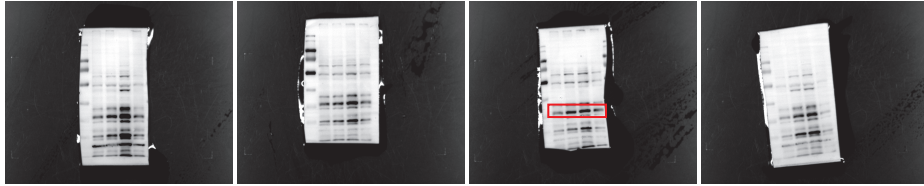

HGFα-Actin

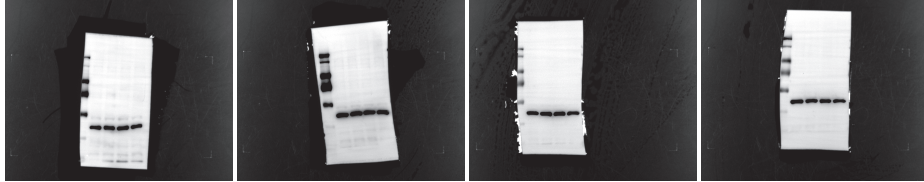

MMP9

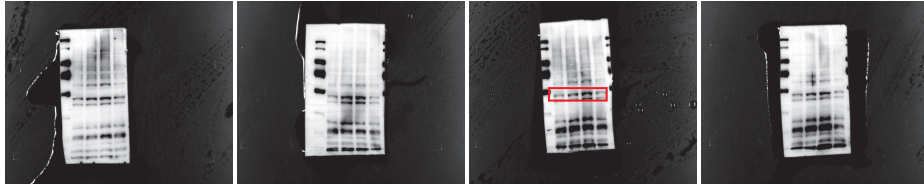

MMP9-Actin

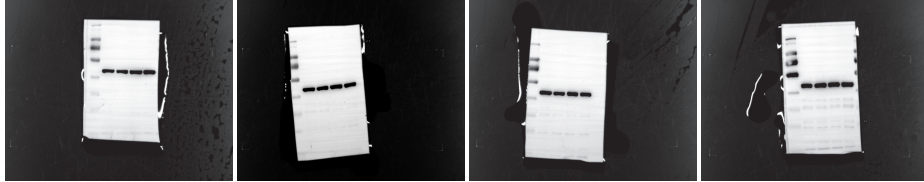

PDGFβ

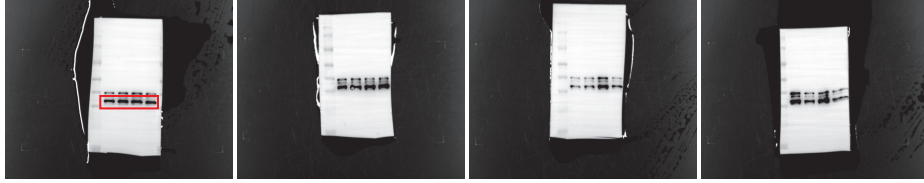

PDGFβ-Actin

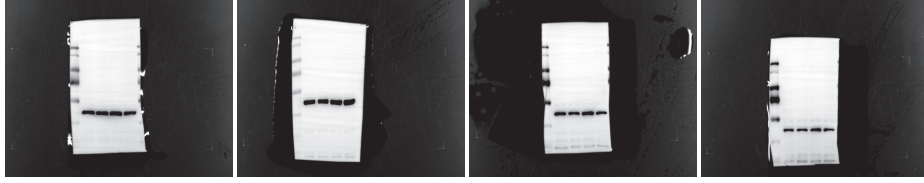

Supplement: Supplementary file 5 — Supplementary Information 2. [file 41598_2023_39473_MOESM5_ESM.pdf]
